# Acetylation of the KXGS motifs in tau is a critical determinant in modulation of tau aggregation and clearance

**DOI:** 10.1093/hmg/ddt402

**Published:** 2013-08-19

**Authors:** Casey Cook, Yari Carlomagno, Tania F. Gendron, Judy Dunmore, Kristyn Scheffel, Caroline Stetler, Mary Davis, Dennis Dickson, Matthew Jarpe, Michael DeTure, Leonard Petrucelli

**Affiliations:** 1 Mayo Clinic, 4500 San Pablo Road, Jacksonville, FL 32224, USA; 2 Acetylon Pharmaceuticals, Inc., 70 Fargo Street, Boston, MA 02210, USA

## Abstract

The accumulation of hyperphosphorylated tau in neurofibrillary tangles (NFTs) is a neuropathological hallmark of tauopathies, including Alzheimer's disease (AD) and chronic traumatic encephalopathy, but effective therapies directly targeting the tau protein are currently lacking. Herein, we describe a novel mechanism in which the acetylation of tau on KXGS motifs inhibits phosphorylation on this same motif, and also prevents tau aggregation. Using a site-specific antibody to detect acetylation of KXGS motifs, we demonstrate that these sites are hypoacetylated in patients with AD, as well as a mouse model of tauopathy, suggesting that loss of acetylation on KXGS motifs renders tau vulnerable to pathogenic insults. Furthermore, we identify histone deacetylase 6 (HDAC6) as the enzyme responsible for the deacetylation of these residues, and provide proof of concept that acute treatment with a selective and blood–brain barrier-permeable HDAC6 inhibitor enhances acetylation and decreases phosphorylation on tau's KXGS motifs *in vivo*. As such, we have uncovered a novel therapeutic pathway that can be manipulated to block the formation of pathogenic tau species in disease.

## INTRODUCTION

Hyperphosphorylated tau (p-tau) is the primary component of neurofibrillary tangles (NFTs), a pathological hallmark of several neurodegenerative diseases including Alzheimer's disease (AD), frontotemporal dementia with parkinsonism associated with chromosome 17, progressive supranuclear palsy, corticobasal degeneration and chronic traumatic encephalopathy, which has recently gained significant attention due to sports- and military-related injuries (1–4). The threshold between preclinical AD and the onset of cognitive symptoms is marked by the increasing burden of NFT pathology, and the accumulation of aggregated p-tau appears to be the primary mediator of cognitive dysfunction (5). As patients progress to severe dementia, the burden of tau pathology rises to peak levels (5). Furthermore, the classification of AD cases into subtypes and the resulting clinical phenotype is ultimately determined by the localization and distribution of tau pathology in the hippocampus and association cortices (6). Therefore, the focus of drug discovery efforts has now shifted towards the prevention of abnormal accumulation of p-tau to slow the progression of AD, but the field has yet to validate a high-value therapeutic pathway to target.

Based on our recent discovery that expression of histone deacetylase 6 (HDAC6) positively correlates with tau burden, while a decrease in HDAC6 activity or expression promotes tau clearance (7), we further investigated the basis of this relationship in the current study. Of note, HDAC6 activity is upregulated in hippocampal neurons exposed to Aβ_1–42_ (8), as well as in the brain of transgenic mice modeling AD amyloidosis (Tg6799 mice) (8), which have been shown to accumulate p-tau (9). HDAC6 levels have also been shown to be elevated in the brains of patients with AD (10,11). In addition, loss of HDAC6 restored cognitive function without impacting Aβ plaque burden in a transgenic mouse model of AD (12), and also rescued the abnormal behavioral phenotype in a *Drosophila* model of tauopathy (13). Collectively, these findings suggest that Aβ-mediated stimulation of HDAC6 deacetylase activity could contribute to the development and progression of tau pathology, and that decreasing HDAC6 activity may interrupt this cycle and prevent the detrimental physiological consequences of tau pathology.

It is thus of particular interest that tau itself is a substrate of HDAC6 (14), which prompted us to critically evaluate the impact of HDAC6-mediated deacetylation on tau biology. Our data demonstrate that HDAC6 regulates the acetylation of tau on KXGS motifs (consisting of KIGS and KCGS motifs), which are localized within tau's microtubule-binding domain and play a crucial role in tau's ability to bind and stabilize microtubules (15–18). In addition, we detect a competitive relationship between acetylation and phosphorylation on this motif, such that acute administration of a selective HDAC6 inhibitor simultaneously blocks phosphorylation and increases acetylation of tau at these crucial KXGS motifs in mice. We also observe a decrease in tau aggregation following its acetylation on KXGS motifs *in vitro*. Furthermore, KXGS motifs are hypoacetylated and hyperphosphorylated in patients with AD, as well as a progressive mouse model of tauopathy (rTg4510), verifying that modulation of this site is linked to disease. Given that tau species phosphorylated on KXGS motifs accumulate in NFTs (19), fail to bind and stabilize microtubules (15) and are also primed for phosphorylation by other kinases (20,21), our results show that augmenting acetylation of KXGS motifs on tau is a promising approach to slow or prevent disease progression in tauopathies.

## RESULTS

### HDAC6-mediated deacetylation of tau's KXGS motifs regulates aggregation potential

**Figure 1.**
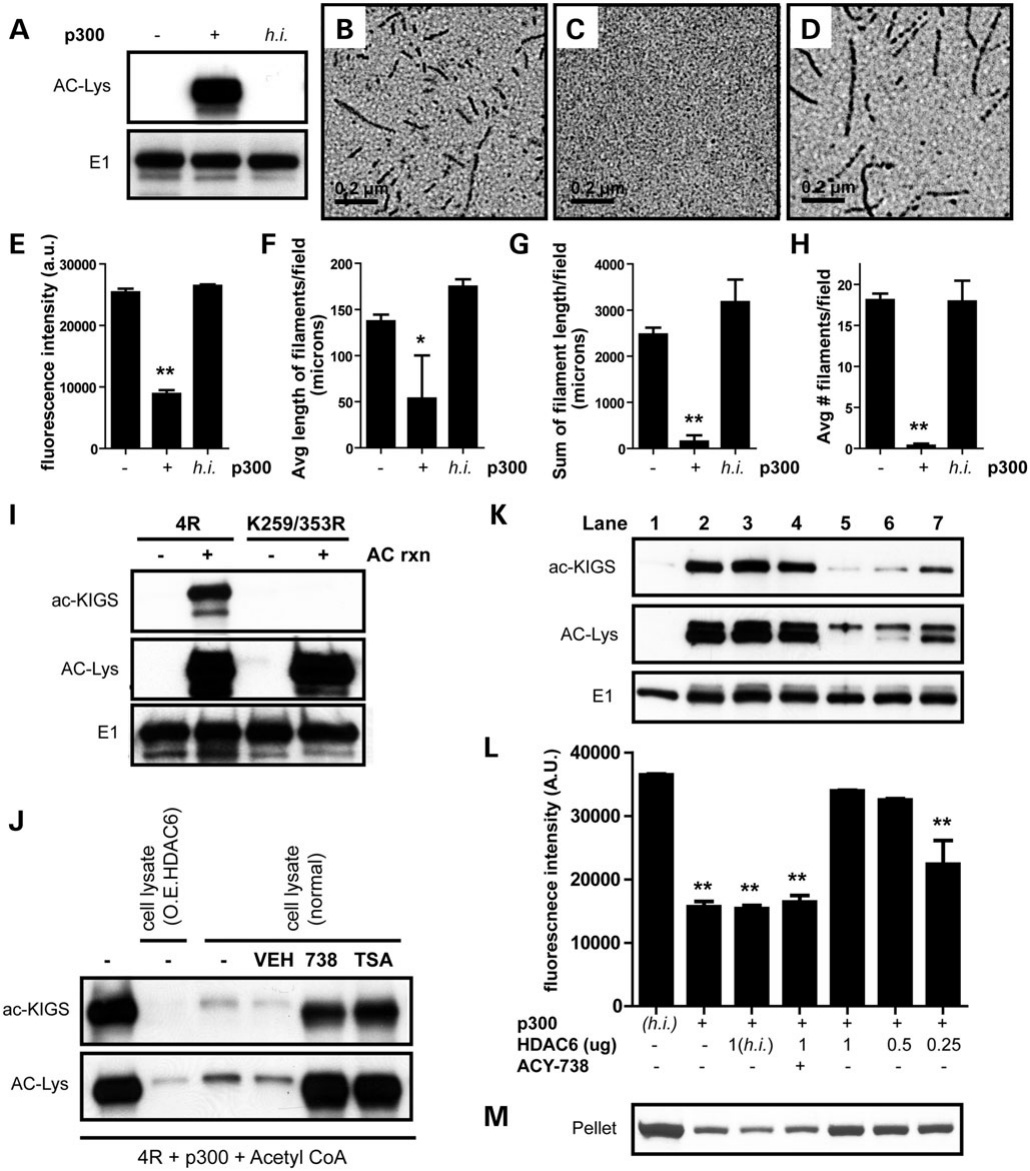
HDAC6 deacetylates KIGS motifs and modulates tau filament assembly. (**A**) Acetyl CoA and recombinant tau were incubated in the absence or presence of active or h.i. p300. Only tau incubated with active p300 showed acetylation by immunoblot. (**B**–**D**) EM revealed that tau readily forms filaments in the absence of p300 (B), but addition of active p300 to the reaction completely prevents tau filament formation (C). The addition of h.i.p300 has no impact on the ability of tau to assemble into filaments (D). (**E**) Quantitation of thioflavin S intensity also confirms that acetylation of tau with active p300 decreases tau polymerization (*F* = 351.8, *P* < 0.0001). (**F**–**H**) Quantitation of EM analysis demonstrated that acetylation decreased the average length of tau filaments per field (*F* = 5, *P* = 0.01) (F), the sum of filament length per field (*F* = 26.7, *P* < 0.0001) (G) and the average number of filaments per field (*F* = 43.4, *P* < 0.0001) (H). (**I**) To characterize our novel antibody (ac-KIGS), recombinant 4R or mutant K259/353R tau protein was acetylated and evaluated by immunoblot, confirming specificity of ac-KIGS for acetylated K259/353. (**J**) 4R tau was acetylated, following the reaction, and incubated with HEK-293T cell lysates either overexpressing HDAC6 or expressing endogenous levels of HDAC6 (normal). In addition, where noted, vehicle (DMSO), ACY-738 or TSA was added along with the cell lysate, and reactions evaluated by immunoblot. (**K**) Recombinant tau was incubated with h.i. p300 (lane 1) or active p300 (lanes 2–7). Following acetylation, h.i. (lane 3) or active HDAC6 (lanes 4–7), and ACY-738 (lane 4) were added as indicated, and reactions were assessed by immunoblot. (**L**) Tau polymerization induced by dextran sulfate was detected by thioflavin S. (**M**) Pelleting analysis was utilized to confirm aggregation. All data are presented as mean ± SEM. Scale bar in (B)–(D) is equal to 0.2 μm. **P < 0.001, *P < 0.05.

We previously demonstrated that overexpression of HDAC6 promotes the accumulation of p-tau, whereas lowering HDAC6 stimulates p-tau clearance (7). To gain a better understanding of the relationship between the deacetylase HDAC6 and tau, we first examined the impact of acetylation on tau filament assembly. To do so, recombinant full-length tau (4R0N isoform) was acetylated *in vitro* by co-incubation with acetyl CoA and the acetyltransferase p300. We anticipated acetylation with p300 would enhance tau assembly as previously reported with the use of the acetyltransferase CREB-binding protein (14). However, we observed robust inhibition of tau aggregation following acetylation, as detected by thioflavin S (Fig. 1E) and electron microscopy (EM; Fig. 1B, C and F–H; Supplementary Material, Fig. S1). While heat-inactivated (h.i.) p300 failed to promote tau acetylation (Fig. 1A) or block tau polymerization (Fig. 1D–H; Supplementary Material, Fig. S1), we demonstrate active p300 catalyzes tau acetylation and prevents tau assembly in a dose-dependent manner (Supplementary Material, Fig. S2A and B). In an effort to identify the key differences between our findings and a previous report (14), we tested a number of different assay conditions, including buffers, enzymes and filament inducers. However, despite testing several assay conditions, we consistently observe a reduction in tau filament assembly following acetylation (Supplementary Material, Fig. S3).

To further investigate the consequence(s) of acetylation on tau, we evaluated the amino acid residues reported to be acetylated by p300, and found all four KXGS motifs in tau's microtubule-binding domain can be modified by acetylation (22). Given that tau's KXGS motifs are known to be critical to tau function (15–18), we sought to assess the impact of acetylation on these motifs. As such, we first generated a novel antibody (ac-KIGS) designed to specifically recognize acetylated-lysine residues within tau's KIGS motifs. To assess specificity of the antibody, we performed an *in vitro* acetylation reaction with either 4R tau or mutant K259/353R 4R tau, containing a mutation in the lysine residue of the first and last KXGS motifs to prevent acetylation at these sites. As shown in Figure 1I, detection with an acetylated-lysine (ac-Lys) antibody confirms that both 4R and mutant tau are acetylated, while the E1 antibody confirms that total tau levels are equal. However, our ac-KIGS antibody detected acetylated wild-type (WT) 4R tau, but not acetylated mutant tau, confirming its specificity for the K259 and K353 sites (Fig. 1I).

Next, to determine the extent to which HDAC6 regulates the acetylation of tau on the KIGS motifs, we performed the *in vitro* acetylation reaction with 4R tau, followed by the addition of cell lysates to the reaction where noted (Fig. 1J). A more dramatic loss of acetylation was observed following the addition of lysates from cells overexpressing HDAC6, though addition of lysates expressing only endogenous HDAC6 still promoted deacetylation of KIGS motifs, albeit to a lesser extent (Fig. 1J). Adding either ACY-738, tubacin, tubastatin A, M344 or BML281 (specific HDAC6 inhibitors), or TSA (trichostatin A; pan-HDAC inhibitor), to the sample blocked the deacetylation of tau upon addition of cell lysates (Fig. 1J; Supplementary Material, Fig. S4). These results, in combination with our finding that MS275 (Class 1 HDAC inhibitor) does not block the deacetylation of tau on the KIGS motifs, confirm that the effect was due to HDAC6 activity (Fig. 1J; Supplementary Material, Fig. S4). Given that SIRT1 has recently been implicated as a tau deacetylase (22), we also tested EX527 (specific SIRT1 inhibitor), AGK2 (specific SIRT2 inhibitor) and nicotinamide (pan-SIRT inhibitor) in our assay, but were unable to block the deacetylation of KIGS motifs (Supplementary Material, Fig. S4), indicating that SIRT1 is not the deacetylase for tau's KIGS motifs.

To further validate that HDAC6 regulates the acetylation of tau on KIGS motifs, we again performed an *in vitro* acetylation reaction with p300 (Fig. 1K, lanes 2–7), using h.i. p300 (Fig. 1K, lane 1) as a negative control. In agreement with our results above, p300 increased acetylation of tau on KIGS motifs, and decreased tau polymerization (Fig. 1K, lane 2). In contrast, addition of recombinant HDAC6 both decreased acetylation of tau's KIGS motifs, and increased tau assembly in a dose-dependent manner (Fig. 1K–M, lanes 5–7). Furthermore, addition of the HDAC6-selective inhibitor ACY-738 (Fig. 1K–M, lane 4) prevented HDAC6-mediated deacetylation of tau on the KIGS motifs, and also blocked the increase in tau polymerization. As an additional control, we verified that h.i. HDAC6 influences neither the acetylation nor assembly of tau (Fig. 1K–M, lane 3). These results demonstrate that HDAC6 directly modulates the acetylation of tau by specifically deacetylating KIGS motifs. In addition, we demonstrate that tau acetylation on KIGS motifs inversely correlates with the ability of tau to polymerize.

### Acetylation of all four KXGS motifs regulates tau assembly

**Figure 2.**
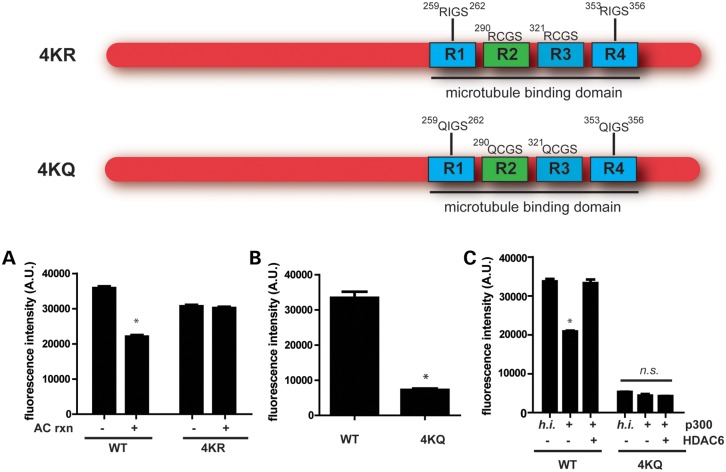
Acetylation of KXGS motifs regulates tau assembly. (**A**) WT or K259/290/321/353R (4KR) mutant proteins were acetylated and incubated with dextran sulfate to promote polymerization, which was detected by thioflavin S. (**B**) WT or K259/290/321/353Q (4KQ) mutant proteins were incubated with dextran sulfate, and tau assembly was detected with thioflavin S. (**C**) WT or 4KQ proteins were acetylated and incubated with HDAC6 where indicated. Dextran sulfate was used to promote tau polymerization, which was detected by thioflavin S. All data are presented as mean ± SEM. *P < 0.001.

To further evaluate the impact of acetylation specifically on KXGS motifs, we introduced either lysine (K) to arginine (R) mutations to prevent acetylation but maintain the charge of the residue or lysine (K) to glutamine (Q) mutations to mimic acetylation on these sites. We thus generated K259/290/321/353 (4KR or 4KQ) mutant proteins, thereby removing all four KXGS motifs. Although acetylation decreased fibrillization of WT full-length tau, acetylation had no effect on filament assembly of the 4KR mutant (Fig. 2A). These results show that the ability of acetylation to decrease tau assembly is dependent on acetylation of the KXGS motifs. In agreement, mimicking acetylation on all four KXGS motifs completely blocked tau assembly (Fig. 2B; Supplementary Material, Fig. S5), providing additional evidence that the ability of acetylation to decrease tau polymerization is dependent upon acetylation of the KXGS motifs.

To assess whether the increase in tau polymerization following addition of HDAC6 is due to modulation of acetylation on KXGS motifs, we acetylated WT or 4KQ tau, and subsequently added HDAC6 to the reaction where noted (Fig. 2C). As previously observed, the assembly of WT tau decreased following acetylation, while HDAC6 reversed the inhibitory effect of acetylation on polymerization. However, the 4KQ tau variant, which is less prone to aggregation, was completely resistant to modulation by either p300-mediated acetylation or HDAC6-mediated deacetylation (Fig. 2C), demonstrating that HDAC6 increases tau polymerization by promoting the deacetylation of KXGS motifs.

### Acetylation and phosphorylation compete to modify KXGS motifs

Given that the KXGS motifs contain acetylation sites critical to inhibiting tau aggregation, as well as serine residues that are sites of phosphorylation, we hypothesized that acetylation may disrupt this motif and prevent subsequent phosphorylation. Notably, the phosphorylation of tau on KXGS motifs by the kinase Par-1/MARK2 is required for tau toxicity in *Drosophila* (21) and is observed at very early stages of NFT formation in AD brain (19). In addition, the synaptic toxicity of oligomeric Aβ is dependent on the phosphorylation of tau's KXGS motifs (23,24), providing additional support for a pathogenic role of this p-tau species. Furthermore, tau species phosphorylated on KXGS motifs are unable to bind and stabilize microtubules (15), and are also not recognized by the HSP chaperone network (25,26), which may explain why this particular p-tau species is prone to accumulation. Collectively, these findings provide strong rationale for the development of a therapeutic strategy aimed at preventing the hyperphosphorylation of tau on the KXGS motifs.

**Figure 3.**
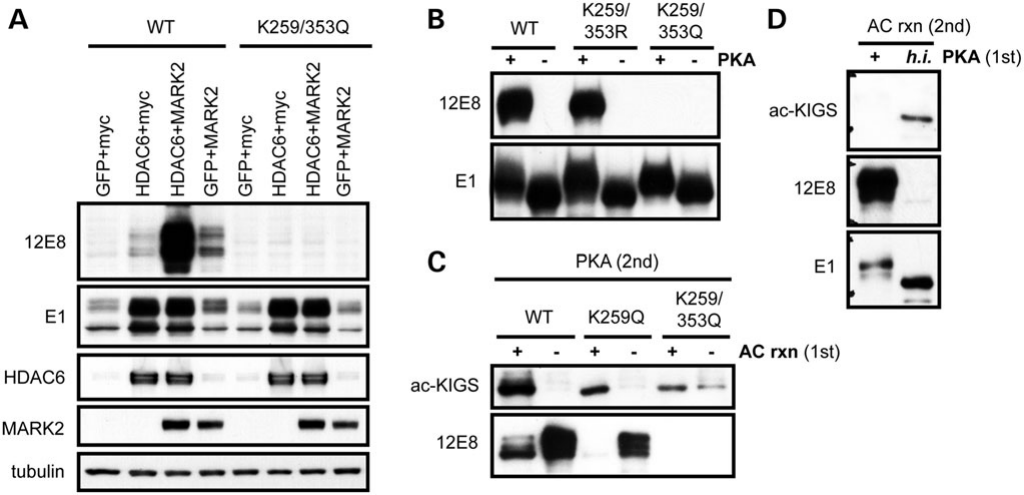
Acetylation and phosphorylation compete to modify KXGS motifs. (**A**) HeLa cells were cotransfected with either 4R or K259/353Q tau, along with GFP, myc vector, HDAC6 or MARK2 as indicated, and cell lysates evaluated by immunoblot. (**B**) Recombinant 4R, K259/353R or K259/353Q mutant tau protein was phosphorylated by PKA, and evaluated by immunoblotting. (**C**) WT, K259Q or K259/353Q mutant tau protein was first incubated in the presence or absence of p300, and subsequently phosphorylated with PKA. (**D**) WT tau protein was first incubated in the presence of active or h.i. PKA, and subsequently acetylated with p300.

Therefore, we sought to assess whether HDAC6, as a deacetylase of the KXGS motifs, would impact the phosphorylation of serine residues within this motif. In agreement with our recent report, in which we demonstrate that HDAC6 activity regulates tau half-life (7), we observed significant tau accumulation under conditions of heightened HDAC6 levels (Fig. 3A). Furthermore, overexpression of the kinase MARK2 increased phosphorylation on KIGS motifs (pS262/356, detected by the antibody 12E8), while co-expression of HDAC6 further enhanced MARK2-mediated phosphorylation at the 12E8 site (Fig. 3A). To determine whether this effect could be blocked by introducing mutations at K259 and K353, site-directed mutagenesis was utilized to generate a construct containing K to Q mutations, mimicking constitutive acetylation. Of note, the K259/353Q mutation completely prevented phosphorylation at the 12E8 site upon MARK2 overexpression (Fig. 3A). We also performed an *in vitro* phosphorylation reaction of recombinant protein using the enzyme PKA to phosphorylate the KXGS motifs as previously described (27,28), given that the only commercially available recombinant MARK2 is inactive. In agreement with results in transfected cells, pseudoacetylation of K259 and K353 (K259/353Q) completely prevented phosphorylation at the 12E8 epitope, though the K to R mutations on these residues (which mimic constitutive deacetylation) had no impact on the ability of PKA to phosphorylate tau *in vitro* (Fig. 3B).

To further investigate the relationship between acetylation and phosphorylation on this motif, we performed an acetylation reaction followed by an *in vitro* phosphorylation reaction of WT, K259Q and K259/353Q mutant tau (Fig. 3C). We observed robust acetylation of the KIGS motifs following acetylation of WT tau. In addition, the single K259Q tau mutant was still positive for ac-KIGS following acetylation, though the signal was reduced as expected, given that the ac-KIGS antibody detects both K259 and K353 residues when acetylated. A slight positive signal was observed with the ac-KIGS antibody for the K259/353Q double mutant in the absence of acetylation, confirming that pseudoacetylation of these residues is effectively mimicking the consequence of acetylation (Fig. 3C). Of note, acetylation decreases the ability of PKA to phosphorylate both the WT and K259Q protein at the 12E8 site; however, the K259/353Q mutant disrupts both KIGS motifs and completely prevents phosphorylation on this epitope (Fig. 3C).

To assess whether phosphorylation similarly blocks acetylation on the KIGS motifs, we performed an *in vitro* phosphorylation reaction with PKA prior to tau acetylation (Fig. 3D). The decrease in acetylation of KIGS motifs following phosphorylation on the 12E8 site verifies that acetylation and phosphorylation compete to modulate tau on the KIGS motifs. Collectively, these results demonstrate that heightened HDAC6 activity facilitates tau hyperphosphorylation on KIGS motifs through deacetylation of these residues. Furthermore, given that loss of HDAC6 also decreases MARK2-mediated phosphorylation on the 12E8 epitope (Supplementary Material, Fig. S6), inhibiting HDAC6 would be predicted to block phosphorylation of KXGS motifs through increased acetylation.

### Inhibition of HDAC6 decreases p-tau levels *in vivo*

**Figure 4.**
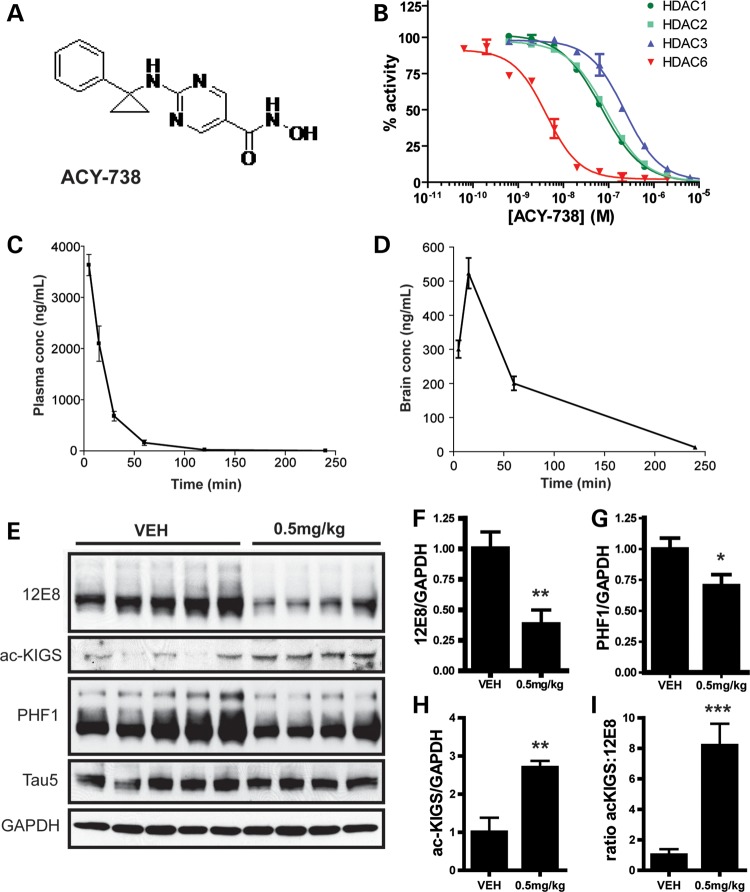
Selective HDAC6 inhibition decreases p-tau levels *in vivo*. (**A**) Structure of ACY-738. (**B**) Specificity of ACY-738 for HDAC6 in comparison to HDAC1-3 was assessed. (**C** and **D**) Non-transgenic mice were injected with ACY-738 (10 mg/kg), and drug levels were assessed in plasma (C) and brain (D) (*n* = 3). (**E**) FVB non-transgenic mice were injected subcutaneously for 3 days with ACY-738 (0.5 mg/kg; *n* = 4–5 mice/group). Brains were collected 1 h after the last injection, and tau levels analyzed by immunoblotting. Blots are representative of three-independent experiments. (**F**) Quantitation of 12E8 immunoreactivity revealed a significant decrease in mice treated with 0.5 mg/kg ACY-738 (*t* = 3.3; *P* = 0.01). (**G**) Quantitation of PHF1 immunoreactivity normalized to GAPDH revealed a significant decrease in ACY-738-treated mice (*t* = 2.3; *P* = 0.05). (**H**) A significant increase in ac-KIGS immunoreactivity was detected in mice injected with ACY-738 (*t* = 3.7; *P* = 0.008). (**I**) The ratio of ac-KIGS to 12E8 is significantly elevated by ACY-738 treatment (*t* = 5.38; *P* = 0.001). All data are presented as mean ± SEM. *P ≤ 0.05, **P ≤ 0.01, ***P ≤ 0.001.

To determine the therapeutic potential of decreasing HDAC6 activity *in vivo*, we used a selective HDAC6 inhibitor, ACY-738 (structure shown in Fig. 4A). This compound inhibits HDAC6 with an IC50 of 2 nm, and is 50- to 125-fold selective versus Class I HDACs (Fig. 4B), and crosses the blood–brain barrier efficiently (Fig. 4C and D). We then administered ACY-738 to non-transgenic mice by subcutaneous injection to assess whether HDAC6 inhibition influences the phosphorylation of tau on KIGS motifs *in vivo.* In agreement with our recent publication (7), we observed a decrease in p-tau levels in mice treated with the HDAC6 inhibitor. Specifically, treatment with 0.5 mg/kg ACY-738, which produced a drug concentration of 7.4 ± 0.6 ng/g in brain, significantly decreased p-tau species recognized by 12E8, as well as PHF1 (pS396/404) (Fig. 4E–G), and also increased acetylation of tau on KIGS motifs (Fig. 4E and H). To more quantitatively measure levels of ac-KIGS and 12E8 in brain homogenates, we also developed sensitive, sandwich immunoassays using the Meso Scale Discovery System (Rockville, MD, USA; Supplementary Material, Fig. S7A and B). As expected, ac-KIGS levels are significantly increased, while 12E8-positive tau levels are significantly decreased in mice treated with ACY-738 (Supplementary Material, Fig. S7C and D). Notably, the ratio of ac-KIGS to 12E8-positive tau species is increased in mice injected with ACY-738 (Fig. 4I; Supplementary Material, Fig. S7E), demonstrating that HDAC6 inhibition modulates this ratio to favor acetylation. Collectively, these results validate that HDAC6 inhibition is an effective strategy to prevent the accumulation of p-tau species (7).

### KIGS motifs of tau are hypoacetylated and hyperphosphorylated in disease

**Figure 5.**
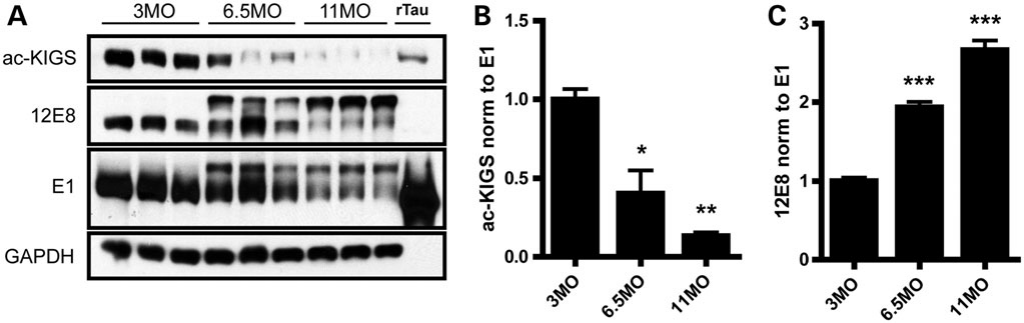
Progressive hypoacetylation of KXGS motifs in rTg4510 mice. (**A**) Levels of ac-KIGS, 12E8 and total human tau (E1) immunoreactivity were evaluated by immunoblotting brain lysates of rTg4510 mice at 3, 6.5 and 11 months of age. Recombinant tau (rTau) that was acetylated *in vitro* served as a positive control for the ac-KIGS antibody. (**B**) Quantitation of ac-KIGS normalized to total tau levels (E1) demonstrated a significant decrease in this tau species with age (*F* = 22.1, *P* = 0.002). (**C**) Quantitation of 12E8 immunoreactivity normalized to total tau levels (E1) revealed a significant increase with aging (*F* = 92.3, *P* < 0.0001). All data are presented as mean ± SEM. *P < 0.05, **P < 0.01, ***P < 0.001.

To evaluate the modification of KIGS motifs in disease, we utilized rTg4510 mice, a well-characterized mouse model of tauopathy (29), and assessed acetylation and phosphorylation of tau using ac-KIGS and 12E8 antibodies, respectively. As expected given the progressive nature of the disease course in this model, pathological 12E8-positive p-tau species increase with age (Fig. 5A and C). Conversely, a dramatic loss of acetylation on KIGS motifs with aging is observed in rTg4510 mice (Fig. 5A and B), confirming that progressive hypoacetylation and hyperphosphorylation of KIGS motifs occurs with disease progression in this model.

**Figure 6.**
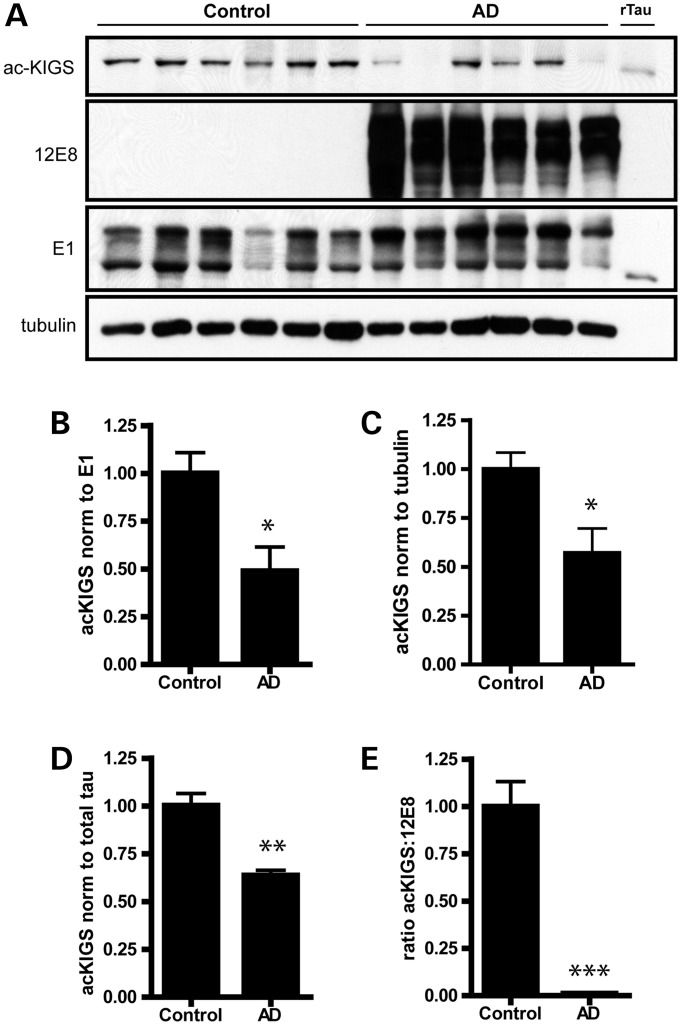
KIGS motifs are hypoacetylated and hyperphosphorylated in AD. (**A**) Levels of ac-KIGS, 12E8 and total human tau (E1) were assessed in frontal cortex from patients with AD compared to control cases. Recombinant tau (rTau) was acetylated *in vitro* and utilized as a positive control for the ac-KIGS antibody. (**B** and **C**) ac-KIGS levels were significantly decreased in AD when normalized to total tau (B; *t* = 3.1, *P* = 0.01), as well as when normalized to tubulin (C; *t* = 2.9, *P* = 0.02). (**D**) MSD sandwich immunoassays were performed to measure ac-KIGS in brain homogenates. Results from the ac-KIGS immunoassay were normalized to results from the total tau assay, which again revealed a decrease in ac-KIGS tau in AD (*t* = 5.3, *P* = 0.0003). (**E**) 12E8 levels were also measured by MSD immunoassay, and the ratio of ac-KIGS to 12E8 is significantly decreased in AD (*t* = 7.5, *P* < 0.0001). All data are presented as mean ± SEM. *P < 0.05, **P < 0.001, ***P < 0.0001.

In order to further assess human disease relevance, we next evaluated the extent to which KIGS motifs are acetylated or phosphorylated in cortical tissue from AD patients compared to control subjects. In agreement with previous work demonstrating that 12E8-positive p-tau species accumulate in NFTs (19), we observed a dramatic shift from a complete absence of 12E8 in control patients, to a significant accumulation of this abnormal p-tau species in AD patients (Fig. 6A). Furthermore, in support of a protective role for acetylation on KXGS motifs, we observed a loss of acetylation on these critical sites in AD patients (Fig. 6A–C). MSD immunoassays were performed to confirm this observation, and a loss of acetylation on KIGS motifs was again observed in patients with AD (Fig. 6D). Therefore, given the decreased ratio of ac-KIGS:12E8 in AD patients (Fig. 6E), and our demonstration that the ratio of ac-KIGS:12E8 is significantly increased in the brain of mice treated with an HDAC6 inhibitor (Fig. 4I; Supplementary Material, Fig. S7E), inhibition of HDAC6 is a promising approach to restore the ac-KIGS:12E8 ratio in disease and alleviate tau burden.

## DISCUSSION

**Figure 7.**
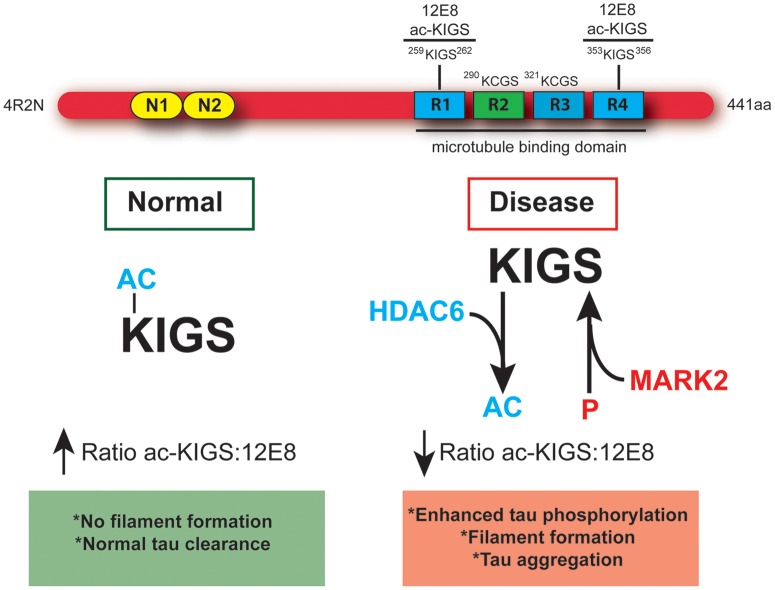
Hypoacetylation of tau's KXGS motifs increases vulnerability to hyperphosphorylation in disease. Schematic diagram depicting full-length (4R2N) tau containing a KXGS motif in each of the four microtubule-binding domain repeats, and illustrating the epitope recognized by the antibodies 12E8 and ac-KIGS. Under normal conditions, acetylation of KIGS disrupts the motif and prevents recognition by the kinase MARK2. This results in a high ac-KIGS:12E8 ratio, normal tau clearance and no filament formation. In disease, hyperactivity of HDAC6 leads to decreased acetylation of KXGS motifs, exposing this motif and increasing susceptibility to phosphorylation by MARK2. This results in a low ac-KIGS:12E8 ratio, enhanced priming of tau for hyperphosphorylation by other kinases, and ultimately leading to tau filament assembly and aggregation.

The results presented in the current report demonstrate that modulation of the acetylation state of tau's KXGS motifs is the foundation of a dynamic relationship between HDAC6 and tau. Specifically, we show that HDAC6 inhibition increases the acetylation of KXGS motifs (Fig. 1; Supplementary Material, Fig. S4), acetylation of KXGS motifs decreases tau polymerization (Figs. 1 and 2; Supplementary Material, Figs. S1–S3) and HDAC6 expression enhances phosphorylation of tau at S262/356 (12E8 epitope), an effect that is blocked by mutation of K259/353 to mimic constitutive acetylation (Fig. 3). Furthermore, we demonstrate that the KXGS motifs are hypoacetylated and hyperphosphorylated in disease (Figs. 5, 6 and 7), and treatment with an HDAC6 inhibitor *in vivo* reverses this ratio to favor acetylation of KXGS motifs (Fig. 4; Supplementary Material, Fig. S7C–E). As summarized in Figure 7, we anticipate that HDAC6 inhibition will restore acetylation on KXGS motifs, thereby blocking subsequent phosphorylation on this epitope. However, given that prior phosphorylation prevents acetylation on KXGS motifs (Fig. 3D), treatment with HDAC6 inhibitors would need to be initiated early in the disease course prior to significant 12E8 accumulation. As such, we envision the use of HDAC6 inhibitors in at-risk patients, identified before the onset of neurological symptoms through improved imaging techniques, as a means to interrupt the development and progression of p-tau accumulation.

HDAC inhibitors have shown great promise in treating a wide variety of diseases. However, to date the safety profile of broad spectrum HDAC inhibitors have precluded their use in chronic, non-oncology indications. Based on published descriptions of HDAC6 knockout mice, loss of HDAC6 activity, in contrast to Class I HDACs, is well tolerated, and HDAC6 appears to be a target with limited liability for deleterious side effects (12,30,31). Therefore, targeting HDAC6 with specific inhibitors could provide a safe means to chronically lower pathological tau levels and block the associated disease cascade. Of importance, HDAC6 expression has been shown to be elevated in AD patients (10,11), and HDAC6 activity is increased in a transgenic mouse model of Aβ amyloidosis, as well as primary hippocampal neurons exposed to Aβ_1−42_, indicating that Aβ may initiate the pathogenic cascade by increasing HDAC6 activity (8). Genetic ablation of HDAC6 in a transgenic mouse model of AD (APPPS1 mice) was shown to alleviate cognitive deficits without impacting Aβ plaque burden (12); although tau levels were not evaluated, APPPPS1 mice develop p-tau neuritic structures (32), which may indicate that the protective effect of loss of HDAC6 is due to effects on tau. This notion is further supported by the finding that loss of endogenous tau alleviates cognitive deficits in APP transgenic mice without affecting Aβ levels (33), which may suggest that Aβ leads to cognitive dysfunction through a cascade that involves HDAC6 upstream of tau. Based on the demonstration that phosphorylation of tau on KXGS motifs is required for synaptic toxicity of Aβ (23,24), therapeutic intervention with an HDAC6 inhibitor to prevent deacetylation and subsequent phosphorylation of tau's KXGS motifs should halt the pathogenic cascade initiated by Aβ prior to the development of synaptic and cognitive deficits.

A similar therapeutic intervention may also aid patients suffering from chronic traumatic encephalopathy, since HDAC6 expression is upregulated in neurons in response to injury (34), and may be involved in disease pathogenesis and generation of toxic tau species following head injury. Given that we observe a decrease in p-tau in non-transgenic mice following HDAC6 inhibition (Fig. 4), we anticipate that treatment of mice with an HDAC6 inhibitor would prevent the development of tau pathology in a mouse model of chronic traumatic encephalopathy, which similarly employ non-transgenic mice (4). Furthermore, the demonstration that HDAC6 expression is relatively high in the hippocampus and cortex in comparison to the cerebellum (12) may provide the basis for the increased susceptibility of these brain regions to develop tau pathology following the initial insult (i.e. Aβ or contact injury).

As the rationale for targeting HDAC6 for the treatment of tauopathies is based on elevating tau acetylation on KXGS motifs, it should be mentioned that recent reports have suggested a pathogenic role for tau acetylation in disease (14,22). However, Min and colleagues utilized the synthetic peptides spanning amino acids 160–182 and 264–287 of the full-length (4R2N isoform) tau sequence to generate acetylated-tau antibodies; therefore, both antigens would fail to produce antibodies against acetylated-KXGS motifs (22). Furthermore, using these acetylated-tau antibodies (referred to as Ab708 and 9AB), SIRT1 was identified as the deacetylase for the acetylation sites contained within amino acids 160–182 and 264–287, while HDAC6 had no effect on the acetylation of these particular residues (22). Given that our current results demonstrate that deacetylation of KXGS motifs is mediated by HDAC6 and not SIRT1 (Fig. 1; Supplementary Material, Fig. S4), this may indicate that the pattern of acetylation on tau is determined by more than one deacetylase. As such, future studies will be required to map the specific acetylation sites regulated by different deacetylases, in order to determine the ultimate consequence(s) of modulators of deacetylase (HDAC6 versus SIRT1) activity on tau function and biology. Regardless, given that lysine residues are unique in their ability to participate in electrostatic and hydrophobic interactions (35), and are also known to play a critical role in tau assembly and toxicity (36–38), it is not surprising that modifying lysine residues by acetylation would have a dramatic impact on tau polymerization.

Given the escalating number of posttranslational modifications identified on tau (14,19,22,39,40), it will be particularly important in future studies to document how posttranslational modifications at specific residues differentially impact tau function and basic biological properties. It is becoming increasingly clear that a complex interplay exists between these various posttranslational modifications, and a greater understanding of how they compete with each other at specific residues is needed. Herein, we demonstrate that acetylation and phosphorylation compete to modify tau's KXGS motifs, and based on the finding that hyperphosphorylation of KXGS motifs is observed early in AD (19), this may indicate that hypoacetylation of KXGS motifs is a key event in the onset of pathology. Although tau acetylation has also been proposed to be pathogenic due to the removal of lysine residues available for ubiquitination (22), insufficient ubiquitination is unlikely to be the cause of tau accumulation in disease given that tau species present in NFTs are already polyubiquitinated (40). In fact, given that ubiquitination would also remove lysine residues available for acetylation, the finding that pathological tau species have been reported to be both ubiquitinated and hyperphosphorylated on KXGS motifs (19,40) would appear to provide additional evidence that decreased acetylation on these critical sites is involved in disease pathogenesis.

Another important implication of the present findings is that while MARK2 phosphorylates KXGS motifs present in each microtubule-binding repeat of tau, KXGS motifs are also present in other microtubule-binding proteins, such as MAP2 and MAP4, and are also substrates of MARK2 (17,41,42). Acetylation and phosphorylation may therefore also compete to modulate the function of all microtubule-binding proteins that contain KXGS motifs, and, by extension, the balance between kinase/phosphatase and acetyltransferase/deacetylase activity may ultimately regulate microtubule dynamics. Given that tubulin acetylation and acetylation of tau on KXGS motifs are both controlled by HDAC6, this would allow for tight regulation of microtubule dynamics and axonal transport. While increased tubulin acetylation promotes microtubule stability (43), increased tau acetylation may allow tau to dissociate from stabilized microtubules, providing molecular motors (such as dynein and kinesin) greater access to microtubules and facilitating axonal transport. Conversely, under conditions of HDAC6 overexpression, tubulin acetylation decreases and microtubules become destabilized; however, the loss of tau acetylation under these same conditions may promote tau–microtubule interactions, leading to increased microtubule stability. Given that phosphorylation within KXGS motifs, which prevents acetylation, has also been reported to release tau from microtubules (15), this event would be expected to uncouple the coordinated regulation of tubulin and tau acetylation, which may further contribute to the pathogenicity of this particular p-tau species.

In conclusion, we identify and validate a novel, therapeutic mechanism designed to block the formation of abnormal p-tau species that mediate neuronal dysfunction and death in disease. Specifically we demonstrate that HDAC6 drives the deacetylation of KXGS motifs, leaving tau vulnerable to pathogenic mechanisms that promote hyperphosphorylation and polymerization. Furthermore, selective HDAC6 inhibition shifts the ratio between acetylation and phosphorylation on tau's KXGS motifs to favor acetylation, preventing tau accumulation. As such, the use of HDAC6 inhibitors to alleviate tau burden through the enhanced acetylation on KXGS motifs represents a very promising therapeutic strategy that should be investigated as a potential treatment for AD, as well as other tauopathies, including chronic traumatic encephalopathy.

## MATERIALS AND METHODS

### Chemicals and reagents

ACY-738 was synthesized and provided by Acetylon Pharmaceuticals, Inc. (Boston, MA, USA). We purchased the recombinant proteins p300 (Enzo Life Sciences Inc., Farmingdale, NY, USA), PKA (New England Biolabs, Ipswich, MA, USA) and HDAC6 (BPS Biosciences, San Diego, CA, USA). We also purchased phosphatase inhibitor cocktails II and III, nicotinamide, TSA, DNAase, chremophor, acetyl CoA, dextran sulfate (MW 6500–10 000 Da), thioflavin S, and Coomassie blue from Sigma-Aldrich (St. Louis, MO, USA). In addition, we purchased dithiothreitol (DTT) and DMSO (Fisher Scientific, Pittsburgh, PA, USA), as well as PIC and isopropyl 1-thio-d-galactopyranoside from EMD Millipore (Billerica, MA, USA).

### Primary antibodies

Anti-ac-KIGS antibody was generated by immunizing a rabbit with two synthetic, acetylated-tau peptides spanning residues 255 and 266 (NVKS[ac-K]IGSTENL and NVK[pS][ac-K]IGSTENL), to ensure that phosphorylation on S258 would not prevent the ac-KIGS antibody from detecting acetylated K259. The antisera collected from the animal was then immunodepleted for the unmodified peptide, and subsequently affinity-purified (21st Century Biochemicals, Marlboro, MA, USA). PHF1 (1:500; anti-S396/S404 p-tau) was provided by P. Davies (Albert Einstein College of Medicine, New York, NY, USA). 12E8 (anti–pS262/S356) was provided by P. Seubert (Elan Pharmaceuticals, San Francisco, CA, USA). Tau 5 (anti-total tau) was provided by L. Binder (Northwestern University Medical School, Chicago, IL, USA). In addition, we purchased anti-acetyl-lysine (1:1000) and anti-MARK2 (1:1000) from Cell Signaling Technology, Inc. (Danvers, MA, USA), anti-tubulin (1:4000) was purchased from Sigma-Aldrich, anti-HDAC6 (1:1000) was purchased from EMD Millipore and anti-GAPDH (1:10 000) was purchased from Meridian Life Science, Inc. (Memphis, TN, USA). E1 (1:1000; human-specific tau antibody) was generated by our group against amino acid residues 19–33 within exon 1 of human tau (7,20,44). Secondary antibodies were obtained from Jackson ImmunoResearch Laboratories, Inc. (West Grove, PA, USA).

### Expression plasmids

MARK2/Par1-myc was kindly provided by B. Lu (Stanford University School of Medicine, Stanford, CA, USA), while GFP and 4R-V5 tau constructs for mammalian expression were cloned into pcDNA3.1 in our lab (26,44). A human HDAC6 clone in a pCMV SPORT6 vector was purchased from Life Technologies (Grand Island, NY, USA). The QuikChange Mutagenesis Kit (Agilent Technologies, Clara, CA, USA) was utilized to generate K259/353Q, 4KQ (K259/290/321/353Q) and 4KR (K259/290/321/353R) constructs from the 4R-V5 parent, following the manufacturer's protocol. For protein expression in bacteria, 4R0N tau constructs (WT, 4KQ and 4KR) were cloned into the pET30a vector (EMD Millipore). The integrity of all constructs was confirmed by automated sequencing.

### Protein purification

Human cDNAs encoding either 4R0N WT or mutant tau were subcloned into pET-30a bacterial expression vectors (EMD Millipore) and transformed in Rosetta 2(DE3) pLysS competent cells (EMD Millipore) for expression. Overnight cultures were grown to saturation, and these were used at 1/100 to inoculate larger cultures in the morning. The cells were then grown in Luria Broth at 37°C in a shaker rotating at 220 rpm until the OD600 reached 0.5 absorbance units. Tau expression was then induced with 0.5 mm isopropyl 1-thio-d-galactopyranoside for 4 h, and the cells were collected by centrifugation and washed in 1× TBS before storage at −80°C. The cell pellets were resuspended in lysis buffer (100 mm Tris, pH 8, 50 mm NaCl, 1 mm MgCl_2_, 1 mm PMSF, 1 mm DTT, 21 U/ml DNAase) and lysed with three freeze–thaw cycles using liquid nitrogen and a tepid water bath. These lysates were centrifuged to remove cellular debris, and NaCl was then added to the supernatants at 500 mm. These were heated at 80°C for 10 min, and this was followed by cooling on ice for 10 min. The precipitates were removed by centrifugation, and the supernatant was loaded onto a 5-ml HiTrap Mono S column (GE Healthcare Life Sciences, Pittsburgh, PA, USA) using the ÄKTA FPLC system (GE Healthcare Life Sciences). The column was washed with 10 column volumes of wash buffer (10 mm HEPES, pH 7.4, 150 mm NaCl), and Tau proteins were eluted with a linear gradient of NaCl at 20 mm/min. Fractions of 1 ml were collected and analyzed by SDS– PAGE and Coomassie blue staining. Fractions containing high concentrations of purified tau protein were pooled and trice dialyzed against 200 volumes of 10 mm HEPES buffer at pH 7.4. Protein concentrations were determined using the BCA Protein Assay Kit (Pierce Biotechnology, Rockford, IL, USA).

### *In vitro* acetylation and phosphorylation assays

Tau protein at a final concentration of 8 µm was incubated with 0.5 µg of human recombinant p300 in buffer A (10 mm HEPES, pH 7.4, 50 mm NaCl, 1.5 mm MgCl_2_, 0.5 mm DTT, 2.5 mm EGTA, 0.1 mm EDTA) with 125 µm acetyl CoA for 4 h at 30°C (30 µl final reaction volume). To heat-inactivate p300, the enzyme was heated at 95°C for 10 min. For the PKA phosphorylation reaction, 8 µm of recombinant tau was incubated with 0.3 U of active or h.i. (95°C for 10 min) PKA per pmol of Tau at 37°C for 4 h in buffer A with 2 mm ATP.

### *In vitro* deacetylation assays

HEK-293T cells were transfected with human HDAC6 or mock plasmid, and 24 h after transfection were lysed in buffer A, sonicated briefly and centrifuged at 16 000 × *g* for 15 min at 4°C. Protein concentration was determined by BCA protein assay, an equal amount of protein was added to the acetylation reaction, vehicle (DMSO) or HDAC6 inhibitors were added to the reaction where noted, and samples were incubated at 30°C for 16 h.

To assess deacetylation with recombinant human HDAC6, following the acetylation reaction, different concentrations of HDAC6 were added to the reaction in the presence or absence of vehicle (DSMO) or ACY-738 (where noted). Heat inactivation of HDAC6 was performed by heating the enzyme at 95°C for 10 min. Reactions were incubated at 30°C for 16 h, and analyzed by immunoblot, tau filament assembly and pelleting analysis.

### Tau filament assembly

Tau polymerization was performed at 37°C in buffer A (10 mm HEPES, pH 7.4, 50 mm NaCl, 1.5 mm MgCl_2_, 0.5 mm DTT, 2.5 mm EGTA, 0.1 mm EDTA) with 0.04 mg/ml of dextran sulfate to induce assembly. In all experiments described, the tau concentration employed was 8 µm (0.33 mg/ml). To study the effect of acetylation on tau polymerization, dextran sulfate was added to the acetylation reaction, and incubated at 37°C for 4 h before analysis.

Tau filament assembly was monitored by fluorescence of thioflavin S binding using a Cary Varian Eclipse Spectrofluorometer (Walnut Creek, CA, USA) with an excitation wavelength of 440 nm and a slit width 10 nm, and emission spectrum collected from 460 to 600 nm and a 10 nm slit width. Measurements were performed at room temperature after incubating 30 μl of tau reaction with 90 μl of 10 µm thioflavin S (7.5 µm final concentration) for 30 min. thioflavin S binding intensity was measured by integrating the curve between the ranges of 460–600 nm using the Cary Eclipse Scan software.

### Pelleting analysis

Samples from assembly reactions were centrifuged at 100 000 × *g* for 75 min at 4°C to separate free and aggregated tau. The pellets containing aggregated and polymerized tau were resuspended in 30 µl 1× sample buffer. Then the pellets were visualized by SDS–PAGE on 10% Tris–glycine gels by Coomassie blue staining.

### Electron microscopy

Reaction samples were diluted 4× in Buffer A, and 10 μl was absorbed onto carbon/formvar-coated 400 mesh copper grids (Electron Microscopy Sciences, Hatfield, PA, USA) for 45 s. These were then stained with 2% uranyl acetate (Electron Microscopy Sciences) for 45 s, and the grids were examined with a Philips 208S electron microscope (Philips, Hillsboro, OR, USA). For quantification, four images were collected randomly at three predetermined coordinate locations that remained fixed throughout these studies. These 12 images were collected at 5000 × *g* magnification, and the average filament number, average filament length and total filament length per field was measured manually using Image J freeware. The length bar was also measured so these lengths could be converted into nanometers.

### Cell culture and transient transfections

HeLa and HEK-293T cells were maintained in Opti-Mem (Life Technologies) supplemented with 10% h.i. FBS (Life Technologies) and 1% PenStrep (Life Technologies) passaged every 3–4 days based on 90% confluency. For plasmid transfections, 2 μg plasmid DNA (1 μg tau construct, 0.5 μg GFP/myc/HDAC6/MARK2) was combined with Lipofectamine 2000 reagent (Life Technologies) for 20 min in 500 μl Opti-Mem (serum-free media), and this mixture was added to cells for 4 h. The transfection mixture was then replaced with fresh complete media.

### Sample preparation and immunoblotting procedure

Cells were harvested in lysis buffer containing 50 mm Tris–HCl (pH 7.4), 274 mm NaCl, 5 mm KCl, 5 mm EDTA, 1% Triton X-100, 1 mm PMSF, protease inhibitor cocktail and phosphatase inhibitor cocktails II and III, followed by sonication. Samples were centrifuged at 16 000 × *g* for 15 min, and a standard BCA Protein Assay Kit (Pierce Biotechnology) performed on the supernatant. Thirty micrograms of protein from each sample were diluted in dH_2_O, 2× Tris–glycine SDS sample buffer (Life Technologies) and 5% beta-mercaptoethanol (Sigma-Aldrich), and heat-denatured for 5 min at 95°C. Samples were run on 10% or 4–20% SDS–PAGE Tris–glycine gels (Life Technologies), and transferred to PVDF membrane (Millipore). Membranes were blocked in 5% non-fat dry milk in TBS/0.1% Triton X-100, and incubated overnight in primary antibody diluted in 5% milk in TBS/0.1% Triton X-100 rocking at 4°C. Membranes were incubated in HRP-conjugated secondary antibodies (1:5000; Jackson ImmunoResearch) for 1 h at room temperature, and detected by ECL (PerkinElmer). Bands were quantified using Scion Image by analyzing pixel density, and protein levels were normalized to the protein loading control.

Human and mice brains were weighed and homogenized in 10× volume of homogenate buffer (50 mm Tris base, pH 8, 274 mm NaCl, 5 mm KCl, 1% Triton X-100, PIC, 1 mm PMSF, phosphatase inhibitor cocktails II and III, 5 mm nicotinamide and 1 μm trichostatin A), sonicated and centrifuged for 15 min at 16 000 × *g* at 4°C to remove cellular debris. Supernatants were collected, protein concentration was determined by BCA assay and samples were processed as described above for immunoblotting.

### HDAC selectivity assays

Potency of each compound was determined using recombinant enzymes and a kinetic method described previously (45). Briefly, HDAC enzymes were diluted to 1.5-fold final concentration in assay buffer and pre-incubated with test compounds for 10 min before the addition of substrate and trypsin. The amount of substrate used for each enzyme was equal to the Michaelis constant (*K*_m_), as determined by a titration curve. The enzymatic reaction was monitored over 30 min for the trypsin catalyzed release of 7-amino-4-methoxy-coumarin after deacetylation of the lysine side chain in the peptide substrate and the linear rate of the reaction was calculated.

### Mice

All animal procedures were approved by the Mayo Institutional Animal Care and Use Committee and are in accordance with the National Institutes of Health Guide for the Care and Use of Laboratory Animals (NIH Publications No. 80-23, revised 1996).

For pharmacokinetic studies with ACY-738, mice were dosed with 10 mg/kg in 0.5% hydroxypropylmethyl cellulose/saline by intraperitoneal injection. Mice were sacrificed at the given times after dosing, and plasma and brain were collected. Compound was extracted and measured by LC/MS/MS using a matrix-matched standard curve to quantitate levels.

For the efficacy study, male FVB (Charles River Laboratories) at 2 months of age were injected (s.c.) with vehicle (5% DMSO, 5% cremophor in saline) or 0.5 mg/kg ACY-738 for 3 days, and euthanized by cervical dislocation 1 h after the last injection. Brains were quickly removed, hemisected and frozen on dry ice. The right forebrain was used for biochemical analysis, and the cerebellum was used to measure drug levels (Agilux Laboratories, Boston, MA, USA). For the aging study, male rTg4510 mice were aged to the indicated time points, euthanized by cervical dislocation, their brains were quickly removed, hemisected and frozen on dry ice. The right forebrain was used for biochemical analysis.

### Human tissue

Human postmortem brain tissue was provided by the brain bank at Mayo Clinic Jacksonville. For these studies, frontal cortex from six control patients (4F, 2M) and six AD patients (3F, 3M) was used. The average age of control patients was 86 years, while the average age of AD patients was 70 years. All AD patients were Braak Stage 6.

### MSD immunoassay

Levels of total tau, ac-KIGS and 12E8 in brain homogenates was measured by a sandwich immunoassay using the Meso Scale Discovery System. Briefly, a 96-well MSD plate was coated with the capture antibody (E1, ac-KIGS or 12E8, respectively), and incubated overnight. The next day, blocking buffer was added to prevent non-specific binding, the plate washed, and standards (recombinant acetylated/phosphorylated tau) or samples were added to the appropriate wells. Following incubation, the plate was washed prior to adding the SULFO-TAG-labeled detection antibody (Tau 5; antibody was conjugated to the SULFO-TAG using a protocol provided by MSD). Tau levels were detected by adding MSD Read Buffer and reading the light emission at 620 nm after electrochemical stimulation using the MSD Sector Imager 2400.

### Data analyses

GraphPad Prism was utilized to perform statistical analyses. Differences between two means were assessed with unpaired two-tailed *t*-tests. Differences among groups with more than two conditions were analyzed using one-way ANOVA, with post hoc analysis using Tukey's multiple comparison tests. *P* < 0.05 was considered statistically significant.

## SUPPLEMENTARY MATERIAL

Supplementary Material is available at *HMG* online.

*Conflict of Interest statement*. M.J. is an employee and shareholder of Acetylon Pharmaceuticals Inc., which provided ACY-738 for the studies described. All other authors have nothing to declare.

## FUNDING

This work was supported by Mayo Clinic Foundation (L.P.), National Institutes of Health/National Institute on Aging [5R01AG026251-04 (L.P.) and AG17216-10JP3 (L.P.)], National Institutes of Health/National Institute of Neurological Disorders and Stroke [R01 NS 063964-01 (L.P.), U01NS065102-1 (L.P.), R01 NS077402 (L.P.)], BrightFocus Foundation A2013546S (L.P.), Sponsored Research – Acetylon Pharmaceuticals Inc. (L.P.), ADRC AG016574 (C.C., Y.C., T.F.G. and M.D.). Funding to pay the Open Access publication charges for this article was provided by Mayo Clinic.

## Supplementary Material

Supplementary Data
