# Supplementary material for: Acetylation of the KXGS motifs in tau is a critical determinant in modulation of tau aggregation and clearance
Source: Hum Mol Genet. 2013 Aug 19;23(1):104–16. doi: 10.1093/hmg/ddt402 (PMC3857946; doi:10.1093/hmg/ddt402)
Supplement: Supplementary Data [file supp_23_1_104__index.html]

Acetylation of the KXGS motifs in tau is a critical determinant in modulation of tau aggregation and clearance — Acetylation of the KXGS motifs in tau is a critical determinant in modulation of tau aggregation and clearance — Supplementary Data 

# Acetylation of the KXGS motifs in tau is a critical determinant in modulation of tau aggregation and clearance

## Supplementary Data

Supplementary Data

**Files in this Data Supplement:**

- Supplementary Data - Doc file
