# Supplementary material for: Acetylation of the KXGS motifs in tau is a critical determinant in modulation of tau aggregation and clearance
Source: Hum Mol Genet. 2013 Aug 19;23(1):104–16. doi: 10.1093/hmg/ddt402 (PMC3857946; doi:10.1093/hmg/ddt402)
Supplement: Supplementary Data [file supp_ddt402_ddt402supp.doc]

**Supplementary Material**


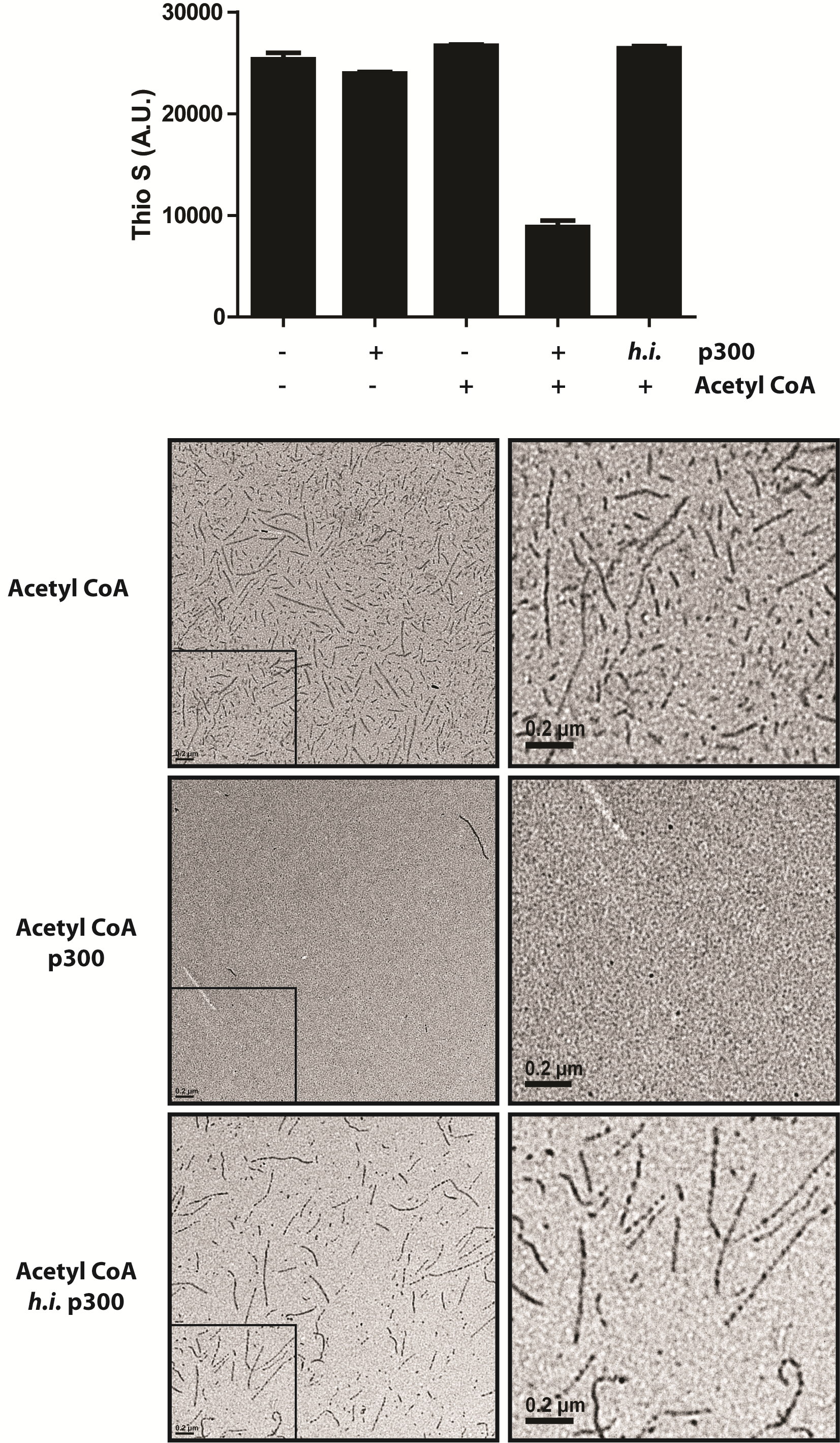


**Supplementary Figure S1. Tau acetylation decreases filament formation.** Recombinant tau (4R0N isoform; 8μM) was incubated in the absence or presence of 125μM Acetyl CoA and 0.5μg active or heat-inactivated (*h.i.*) p300, where noted. Quantitation of Thioflavin S intensity reveals that active p300 and Acetyl CoA are required to decrease tau polymerization. The lack of tau filament formation in the presence of active p300 and Acetyl CoA is also demonstrated by EM.


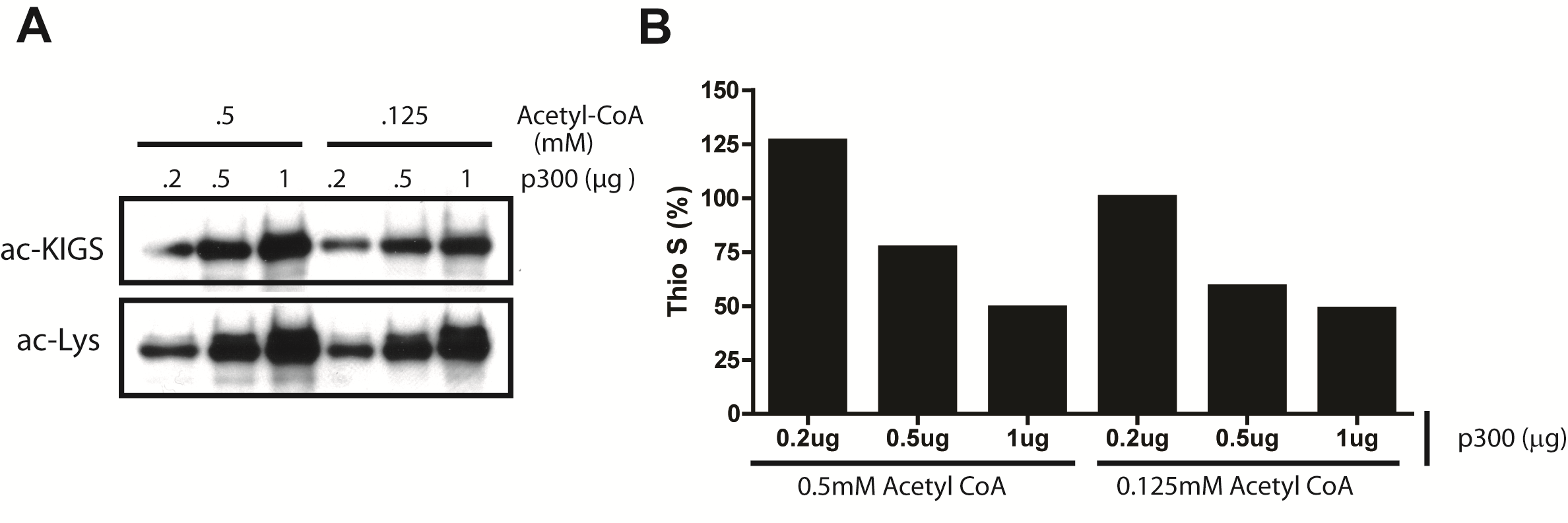


**Supplementary Figure S2. p300 promotes tau acetylation and decreases tau polymerization in a dose-dependent manner.** (A) Recombinant tau (8μM) was incubated with increasing amounts of p300 (0.2μg, 0.5μg, 1μg) at 0.5mM or 0.125mM Acetyl CoA, and the level of total tau acetylation and KXGS acetylation assessed by immunoblot. (B) Quantitation of Thioflavin S confirmed that increasing p300 activity promotes a greater inhibitory effect on tau assembly.


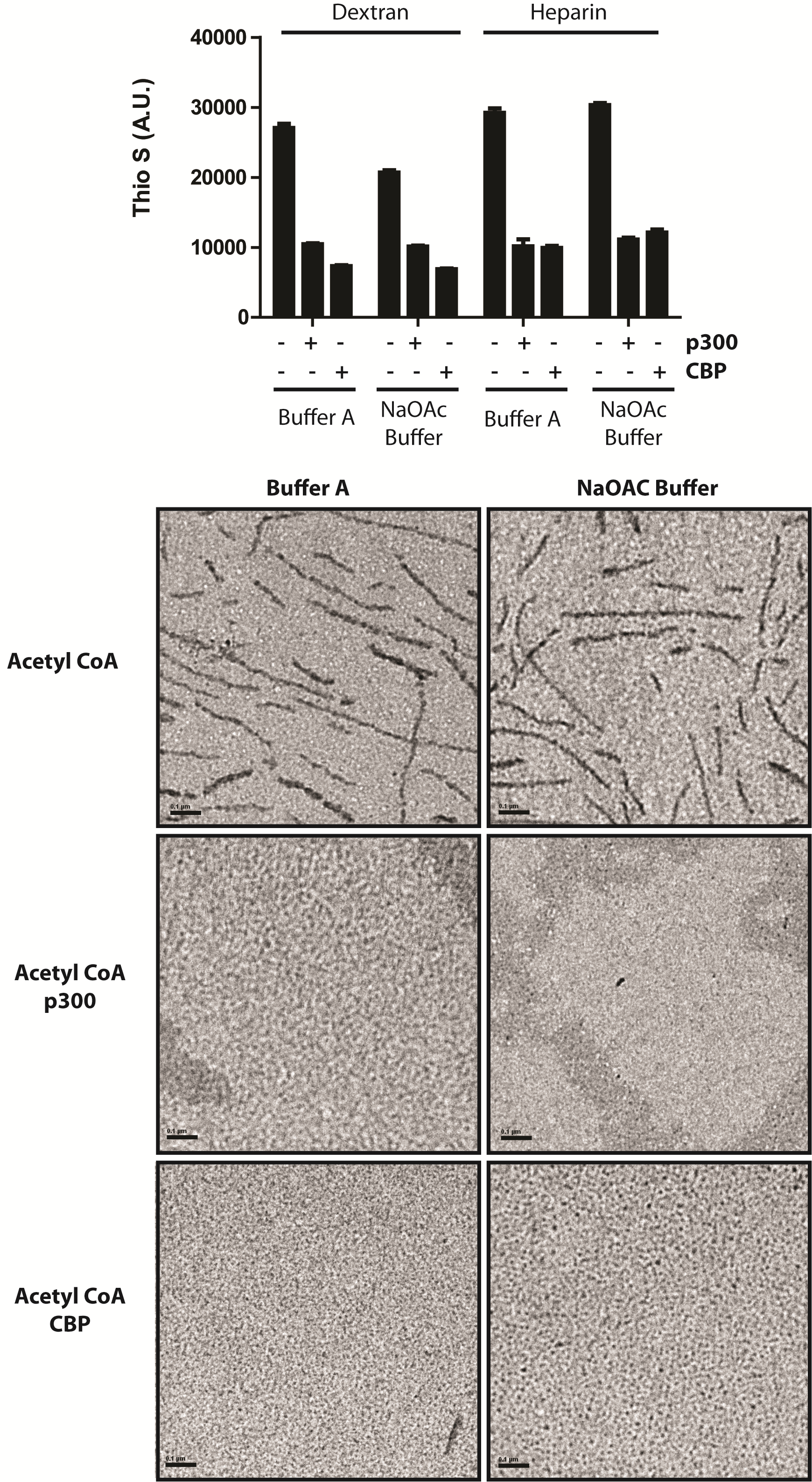


**Supplementary Figure S3. CBP and p300 decrease tau filament assembly.**

**Supplementary Figure S3. CBP and p300 decrease tau filament assembly.** Recombinant tau (8μM) was incubated in either Buffer A or 100mM sodium acetate (NaOAc) buffer in the absence or presence of 0.5μg p300 or CBP acetyltransferase enzymes (final reaction volume 30μL), where noted. The reaction was incubated at 30C for 4hrs, and filament formation subsequently induced with 0.4μg/μL of either dextran sulfate or heparin, followed by incubation at 37C for an additional 4hrs. Thioflavin S (final concentration 7.5μM) was then added to the reaction, incubated for 30 minutes in the dark at ambient temperature, and the fluorescence subsequently measured (excitation at 440nm and emission 460-600nm). The quantitation of Thioflavin S verifies that both p300 and CBP decrease tau filament assembly in either Buffer A or NaOAc buffer, and in the presence of either dextran sulfate or heparin. The lack of filament formation following tau acetylation with either p300 or CBP in both reaction buffers following induction with heparin is confirmed by EM.


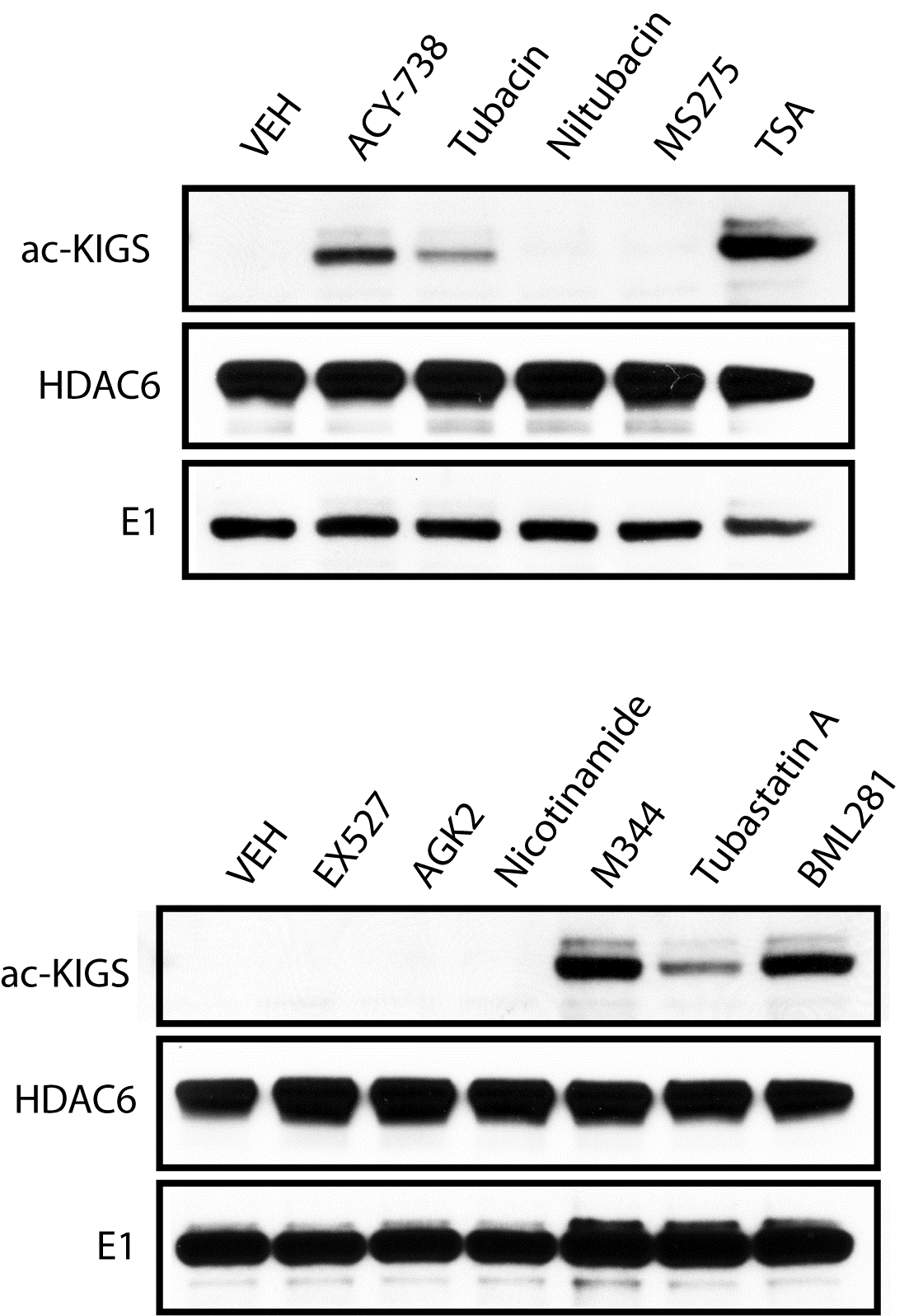


**Supplementary Figure S4. HDAC6 regulates the acetylation of tau’s KXGS motifs.** Recombinant tau (8μM) was acetylated with 0.5μg p300, and following the reaction, incubated with HEK-293T cell lysates transfected with HDAC6. In addition, where noted, vehicle (DMSO), ACY-738 (HDAC6 inhibitor), tubacin (HDAC6 inhibitor), niltubacin (inactive analog of tubacin), MS275 (class I HDAC inhibitor), TSA (pan HDAC inhibitor), EX527 (SIRT1 inhibitor), AGK2 (SIRT2 inhibitor), nicotinamide (pan SIRT inhibitor), M344 (HDAC6 inhibitor), tubastatin A (HDAC6 inhibitor), and BML281 (HDAC6 inhibitor) were added along with the cell lysate. The reactions were incubated at 30C for an additional 16hrs, followed by subsequent evaluation by immunoblot. The results demonstrate that only chemical compounds that inhibit HDAC6 are able to prevent the deacetylation of tau on the KXGS motifs.

**
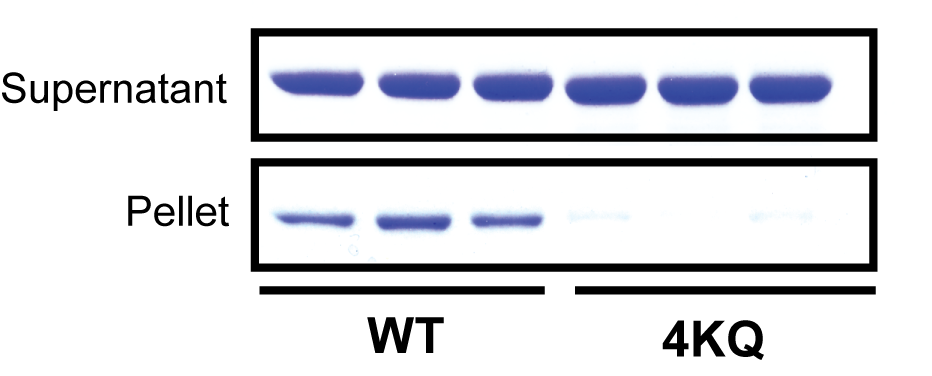
**

**Supplementary Figure S5. Tau construct pseudoacetylated on KXGS motifs does not aggregate.** Pelleting analysis was performed on wild-type (WT) and 4KQ mutant tau protein, and confirmed that the 4KQ tau mutant does not partition to the insoluble pellet.


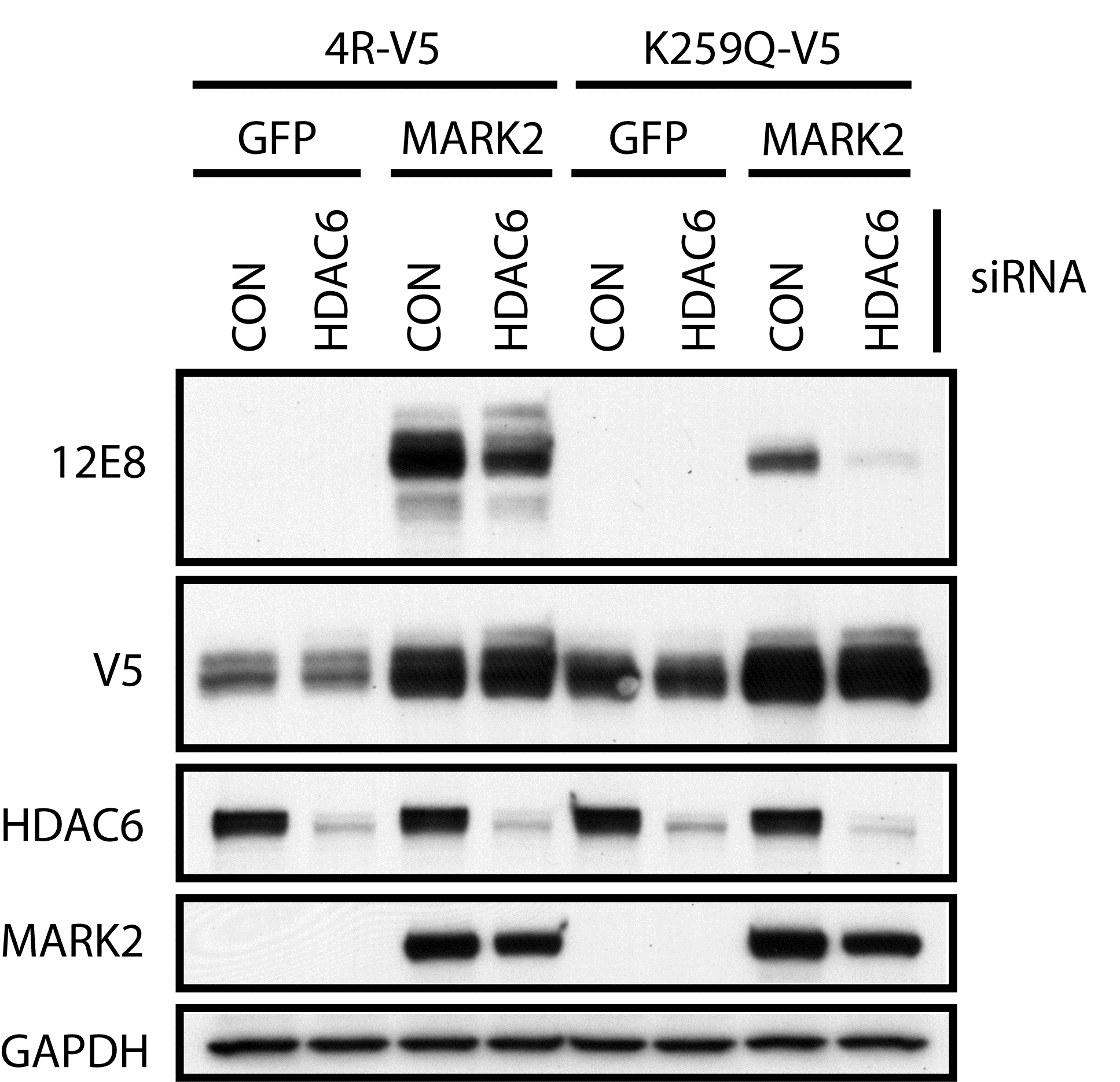


**Supplementary Figure S6. Loss of HDAC6 decreases phosphorylation on KIGS motifs.** Experimentally-verified siControl and siHDAC6 were obtained from Qiagen, and utilized to decrease HDAC6 expression in HeLa cells. Overexpression of the kinase MARK2 increased phosphorylation of 4R tau at the 12E8 site as expected, but loss of HDAC6 decreased phosphorylation at the 12E8 site upon MARK2 overexpression. Introduction of the K259Q mutation further decreased 12E8 in the presence of MARK2, while knockdown of HDAC6 prevents MARK2-mediated phosphorylation at 12E8.


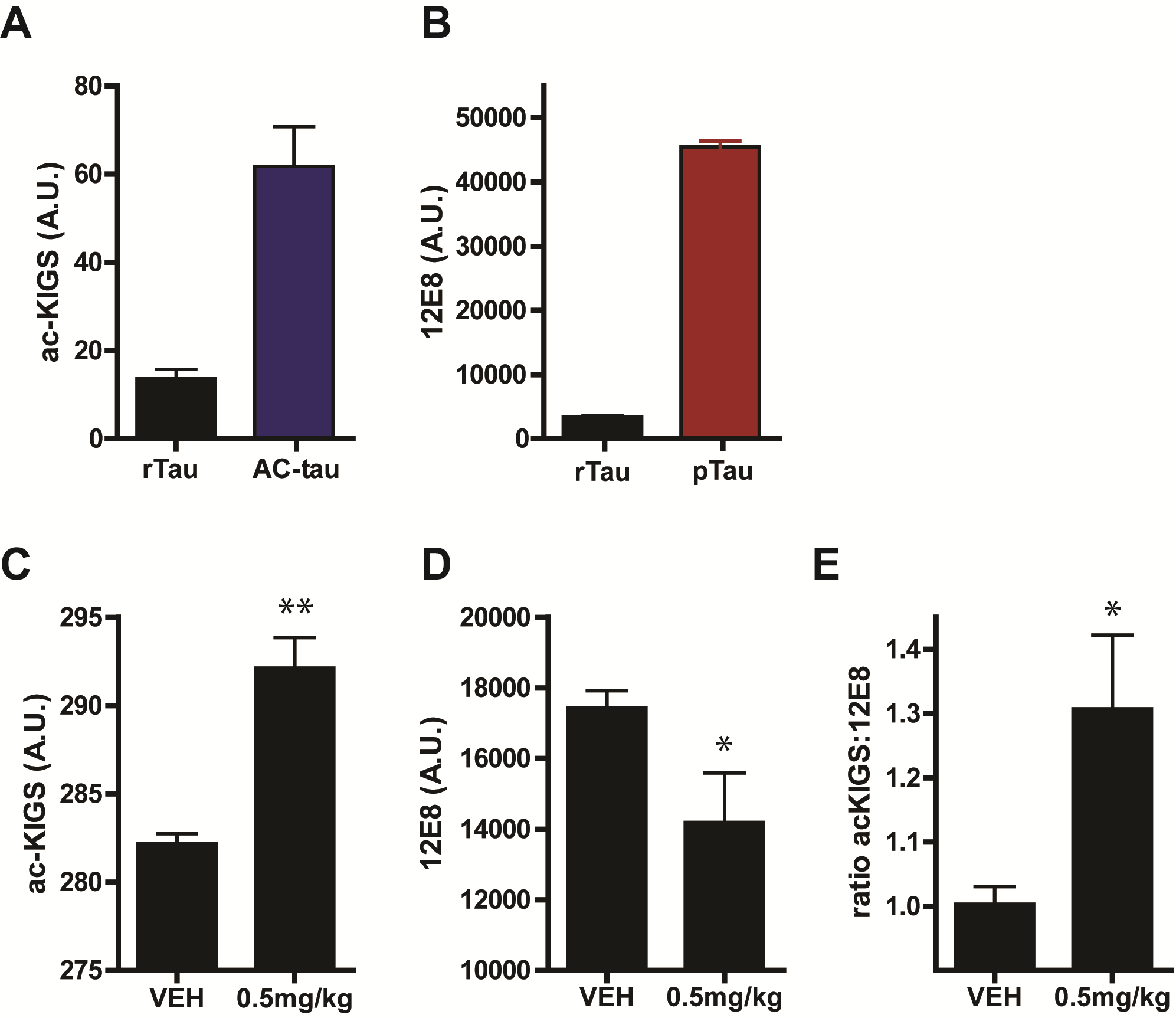


**Supplementary Figure S7. Optimization and validation of MSD immunoassays.** (A) ac-KIGS antibody was coated on the bottom of an MSD plate, and recombinant tau that was either positive (AC-tau) or negative (rTau) for acetylation was added to the well. A total tau antibody (Tau 5) was used as a detection antibody, and the increase in signal observed with AC-tau verifies that the assay is preferentially detecting acetylated tau. (B) For the 12E8 assay, 12E8 was used as the capture antibody, and recombinant tau that was either phosphorylated (pTau) or not (rTau) was added to the well. Tau 5 was again used as the detection antibody, and the increased signal for pTau verifies that the 12E8 assay preferentially detects phosphorylated tau. (C) ac-KIGS levels were significantly elevated in brain homogenates from mice treated with ACY-738 by MSD immunoassay (*t*=6.04, *p*=0.0005). (D) 12E8 levels were significantly decreased in brain homogenates from mice treated with ACY-738 by MSD immunoassay (*t*=2.4, *p*=0.05). (E) MSD sandwich immunoassays revealed an increase in the ratio of ac-KIGS to 12E8 in mice treated with ACY-738 (*t*=2.9; *p*=0.02). All data are presented as mean + SEM. **p*<0.05, ***p*<0.001
